# Editorial: Structural dynamics of membrane proteins, Volume II

**DOI:** 10.3389/fmolb.2022.1037083

**Published:** 2022-10-13

**Authors:** H. Raghuraman, Anja Böckmann

**Affiliations:** ^1^ Crystallography and Molecular Biology Division, Saha Institute of Nuclear Physics, Homi Bhabha National Institute, Kolkata, India; ^2^ Microbiologie Moléculaire et Biochimie Structurale (MMSB), UMR5086 CNRS, Lyon University, Lyon, France

**Keywords:** membrane proteins, mechanosensitive channels, outer membrane proteins (OMP), FTIR difference spectroscopy, lipid-protein interactions

Membrane proteins are crucial elements for numerous cellular functions like electrical excitability, cell-cell communication, ion transport and cellular signalling, and in regulating cellular homeostasis. Further, dysregulation of membrane proteins is associated with various diseases that include heart diseases, neurodegenerative diseases, cancer etc. Considering the fact that approximately two-thirds of the approved drugs target membrane proteins (Bull and Doig, 2015), detailed understanding of the function of membrane proteins is the need of the hour in biomedical research to aid drug discovery. The mechanistic details of a membrane protein function are only possible if the structural dynamics associated with the different functional states is properly understood. Despite the significance and recent advances in solving high-resolution structures, only ∼3% of solved structures in the Protein Data Bank constitutes membrane proteins (Li et al., 2021). This lack of high-resolution structures of membrane proteins can be attributed to several factors like poor expression and extraction, low yield, complexity, and heterogeneity of source membrane along with low success rate of forming well-ordered 3D crystals. Particularly, extracting the membrane proteins from their native hydrophobic environment into a soluble membrane-mimetic milieu in pure, stable, and functional form is the biggest obstacle in obtaining large scale of pure, stable, and functional proteins required for monitoring the structural dynamics using sophisticated biophysical techniques (Brahma and Raghuraman, 2022). Importantly, it should be noted that most of the high-resolution structural studies of membrane proteins are carried out in non-physiological conditions (i.e., in micellar environment) and thereby lack the information on the crucial role of lipid-protein interactions in influencing the membrane protein structure and function (Lee, 2011).

Since the first report on lipid-protein interactions to provide evidence of annular lipids in membranous cytochrome oxidase using electron spin resonance (ESR) spectroscopy (Jost et al., 1973), various sophisticated spectroscopic approaches have effectively been used to decipher how membranes shape the protein structure and thereby its function and vice versa. In case of mechanosensitive ion channels, the relationship between their function and the lateral bilayer pressure of membranes is well documented (Perozo, 2006; Reddy et al., 2019). Crea et al. have presented a novel approach to photoactivate the mechanosensitive channel MscL to one of its sub-conducting states using Azo-PC, a commercially available phospholipid with an azobenzene moiety incorporated into the *sn*-2 acyl chain. In addition, Fourier transform infrared (FTIR) difference spectroscopy of this photomechanical system shows that the light-induced conformational changes in MscL channel are reversible. Similarly, Baserga et al. have used FTIR difference spectroscopy and light activatable lipid analogue Azo-PC to understand how membrane protein activity induces specific molecular changes in lipids by reconstituting several membrane proteins in lipid Nanodiscs. This study demonstrates that the conformational changes in membrane proteins induce a significant local and global perturbation in the collective state of the lipids in the membrane.

Tight junction-forming protein complexes tightly links two neighbouring epithelial or endothelial cells, and *in vitro* characterization of these important proteins are limited compared to other intrinsic transmembrane proteins. Ahlswede et al., has analysed the *cis*- and *trans*-interactions of the tight junction forming protein, human Claudin 7 (Cldn7), a 211 amino acid protein that is expressed in the collecting duct of the kidney, respiratory tract, intestine, and epididymis on the basolateral side of epithelial cells. This is done by heterologously expressing the protein in *E. coli* cells and utilizing membrane-mimetic systems and various sophisticated approaches. Basically, the authors could establish conditions for in-depth analyses of Cldn7 oligomerization within a membrane and the impact of this oligomerization on the formation of tight junctions. Such studies and analyses can help in understanding other tight junction proteins such as Occludins. Another article in this thematic issue deals with the outer membrane proteins (OMP) of Gram-negative bacteria. These β-barrel proteins mostly function as diffusion pores and structural components of the cell wall. Hermansen et al. has characterized one of the smallest Outer membrane proteins namely the OmpX, (M.W. 16.5 KDa), which is involved in regulation of surface adhesion and serum resistance. Here, the authors have used OmpX as a model system to quantify the effects of loop insertions on *in vitro* and *in vivo* OMP folding and stability.

Studies focusing on various classes of membrane proteins in physiologically-relevant membrane environment using sophisticated approaches to monitor functionally-relevant conformational dynamics like site-directed fluorescence (Raghuraman et al., 2019; Chatterjee et al., 2021; Brahma et al., 2022), nuclear magnetic resonance (NMR) (Liang and Tamm, 2016; Opella and Marassi, 2017), high-speed atomic force microscopy (HS-AFM) (Rangl et al., 2019) and cryo-electron microscopy (cryo-EM) (Reddy et al., 2019) are on the rise, which would not only help in validating the existing structures, but also help in understanding the function of membrane proteins in a comprehensive way. We hope that this exciting research field will continue to attract more researchers to understand the intricacies of these complicated yet wonderful biological nanomachines.
